# Paraneoplastic movement disorder due to suspected metastatic Leiomyosarcoma of tongue: A case report

**DOI:** 10.1002/ccr3.8648

**Published:** 2024-03-07

**Authors:** Pradeep Khanal, Pitambar Khanal, Sandip Paudel, Ashbita Pokhrel, Subodh Chapagain

**Affiliations:** ^1^ Department of Internal Medicine Trinity Health Ann Arbor Hospital Ypsilanti Michigan USA; ^2^ Patan Academy of Health Sciences Lalitpur Nepal; ^3^ Institute of Medicine Tribhuvan University Kathmandu Nepal; ^4^ Department of Anatomic and Clinical Pathology William Beaumont Hospital Royal Oak Michigan USA

**Keywords:** case reports, leiomyosarcoma, movement disorder, paraneoplastic syndrome

## Abstract

Paraneoplastic movement disorders, though rare, can be the initial symptoms of malignancies like leiomyosarcoma, as in our case. Clinicians should keep malignancies in their differential diagnosis in cases of unexplained movement abnormalities.

## Graphical abstract

FDG‐avid metastatic pulmonary nodules in a patient with leiomyosarcoma.

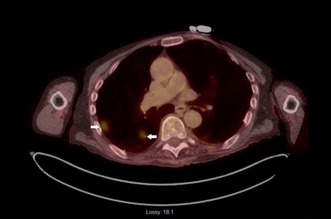

## 1 INTRODUCTION

Paraneoplastic movement disorders (PMD) are uncommon autoimmune non‐metastatic complications of malignancy. They manifest as various movement abnormalities, including chorea, dystonia, opsoclonus‐myoclonus syndrome, cerebellar degeneration, stiff person syndrome, and neuromyotonia and typically occur before tumor diagnosis.^1^, ^2^ It is thought that shared antigens, normally expressed primarily by the nervous system but ectopically expressed by tumors, cause paraneoplastic neurological movement disorders.^2^, ^3^ Presentation of PMD depends upon the affected brain area and frequently overlaps. Common presentations of PMD include ataxia, myoclonus, dyskinesia, chorea, behavioral changes, oculomotor abnormalities, dystonia, and seizures. Treatment includes tumor resection along with other interventions, including steroids, intravenous immunoglobulin, plasma exchange, and immunosuppressants in some cases.

Leiomyosarcoma (LMS) is an aggressive tumor of smooth muscle origin. Only a few cases of LMS with the primary site as the tongue and widespread metastasis have been reported in the literature. The most common presentation of LMS is a mass effect on adjacent organs, and a biopsy of a suspected lesion is needed to make a definitive diagnosis.^4^ Treatment depends on the stage of presentation, with surgical resection being the mainstay of treatment for localized tumors. The goal of management of a metastatic tumor is to control symptoms, decrease tumor bulk, and prolong survival.^4^ Here, we present a case of an 88‐year‐old male with abnormal body movements as the initial presentation of LMS. To the best of our knowledge, this is the first reported case of suspected metastatic LMS of the tongue initially presenting with a movement disorder.

## 2 CASE HISTORY AND EXAMINATION

An 88‐year‐old male with a significant past medical history of atrial fibrillation, hypertension, and hyperlipidemia presented with abnormal movements of the left hand, recurrent falls, and gait instability for 1 month. The symptoms began as sudden jerking movements of the left upper extremity, which later progressed to the right upper extremity and eventually involved the neck. The lower extremities were not affected. Initially, the abnormal movements were infrequent, occurring a few times a day, but progressively worsened over time. Each episode lasted for a few seconds. There was no history of associated sensory loss in the affected area. Additionally, the patient also reported unintentional weight loss of about 10 pounds in the last 3 months.

On examination, his blood pressure was 137/67 mm Hg, heart rate was 55 beats per minute, respiratory rate was 18 per minute, temperature was 98.6 degrees Fahrenheit, and oxygen saturation was 96% on room air. Physical examination showed sudden, involuntary, and jerky movements alternating in bilateral upper extremities and the head, with each episode lasting for a few seconds. Gait instability was also noted on examination. The patient was alert and oriented to time, place, and person. The cranial nerve examination was intact. Strength and sensation were within normal limits.

## 3 METHODS

Laboratory evaluation showed normal hemoglobin, white blood cell count, and platelets. Chemistry panels were within normal limits. Thyroid function tests showed increased free T4 of 1.62 ng/dL (normal: 0.61–1.24 ng/dL) and thyroid stimulating hormone level of 1.22 mcIU/mL (normal: 0.45–5.33 mcIU/mL). Imaging studies, including computed tomography (CT) scan of the head, did not reveal any acute intracranial pathology, and magnetic resonance imaging (MRI) of the brain showed no acute intracranial hemorrhage or infarct, or suspicious intracranial enhancement. The electroencephalogram (EEG) did not show any focal neuronal dysfunction or epileptiform activity. The echocardiogram showed a normal ejection fraction of 63% without any regional wall motion abnormalities and valvular disease.

**FIGURE 1:**
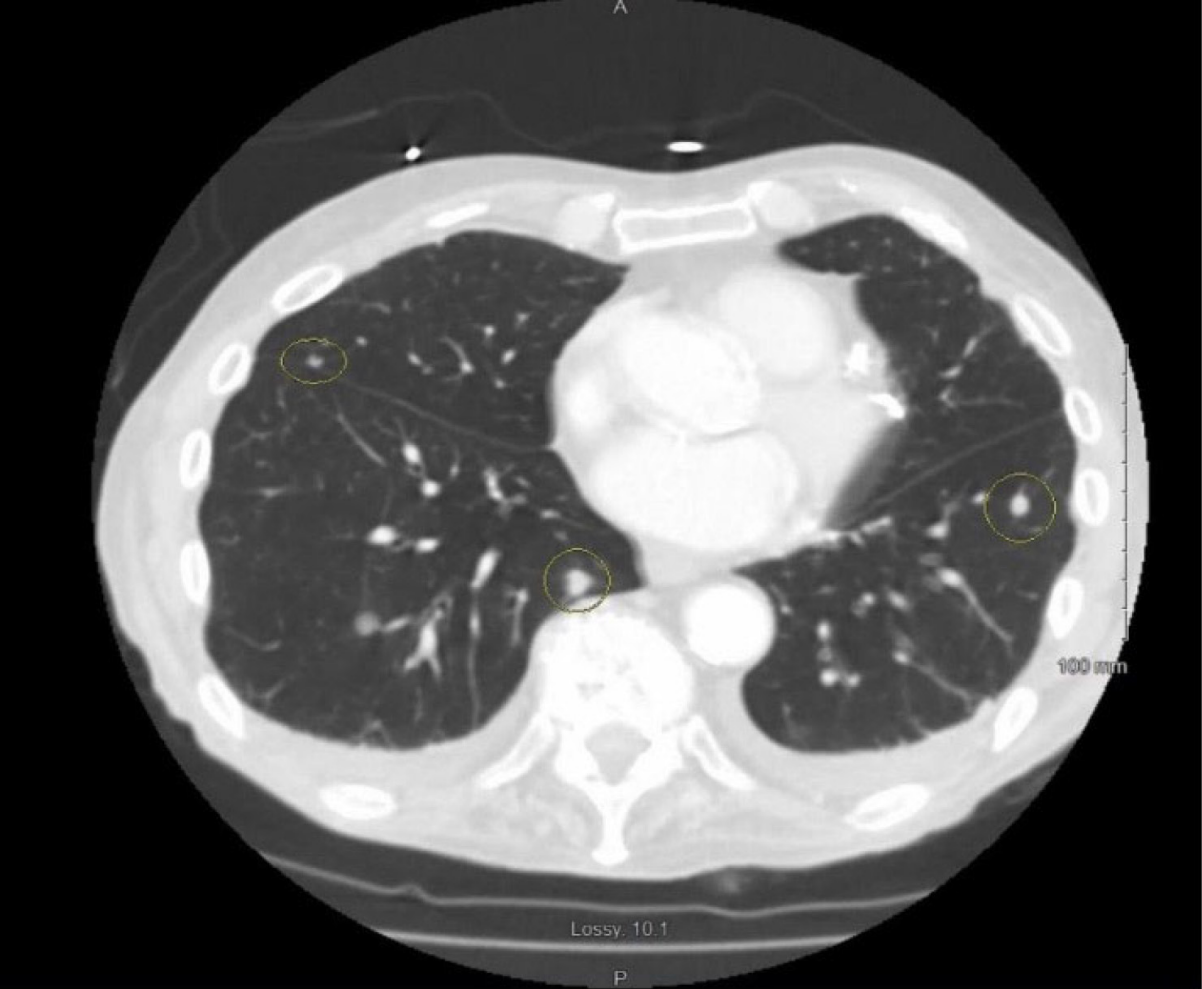
CT chest demonstrating bilateral noncalcified pulmonary parenchymal nodules (circled).

**FIGURE 2:**
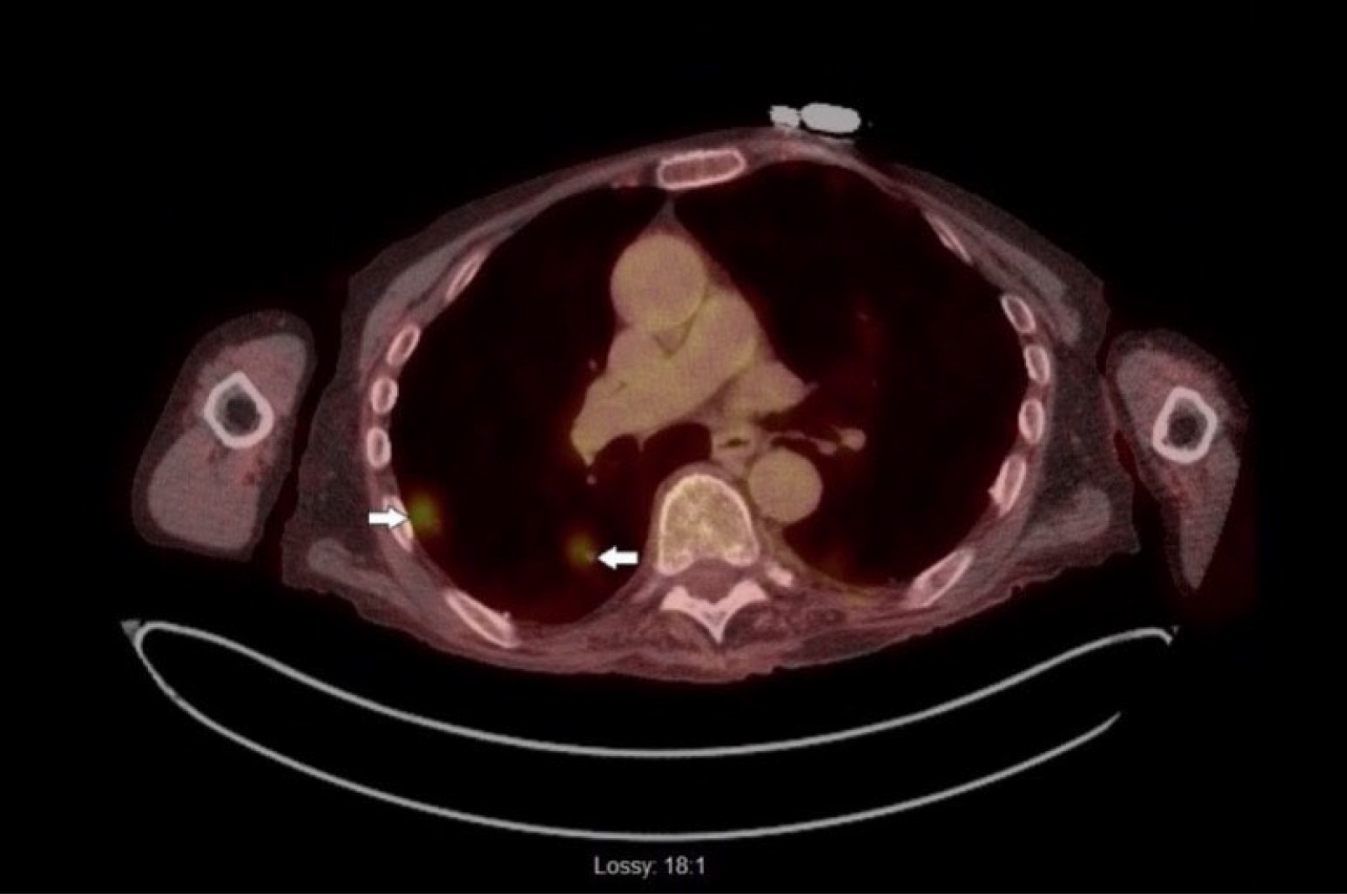
PET CT demonstrating innumerable FDG avid pulmonary nodules.

**FIGURE 3:**
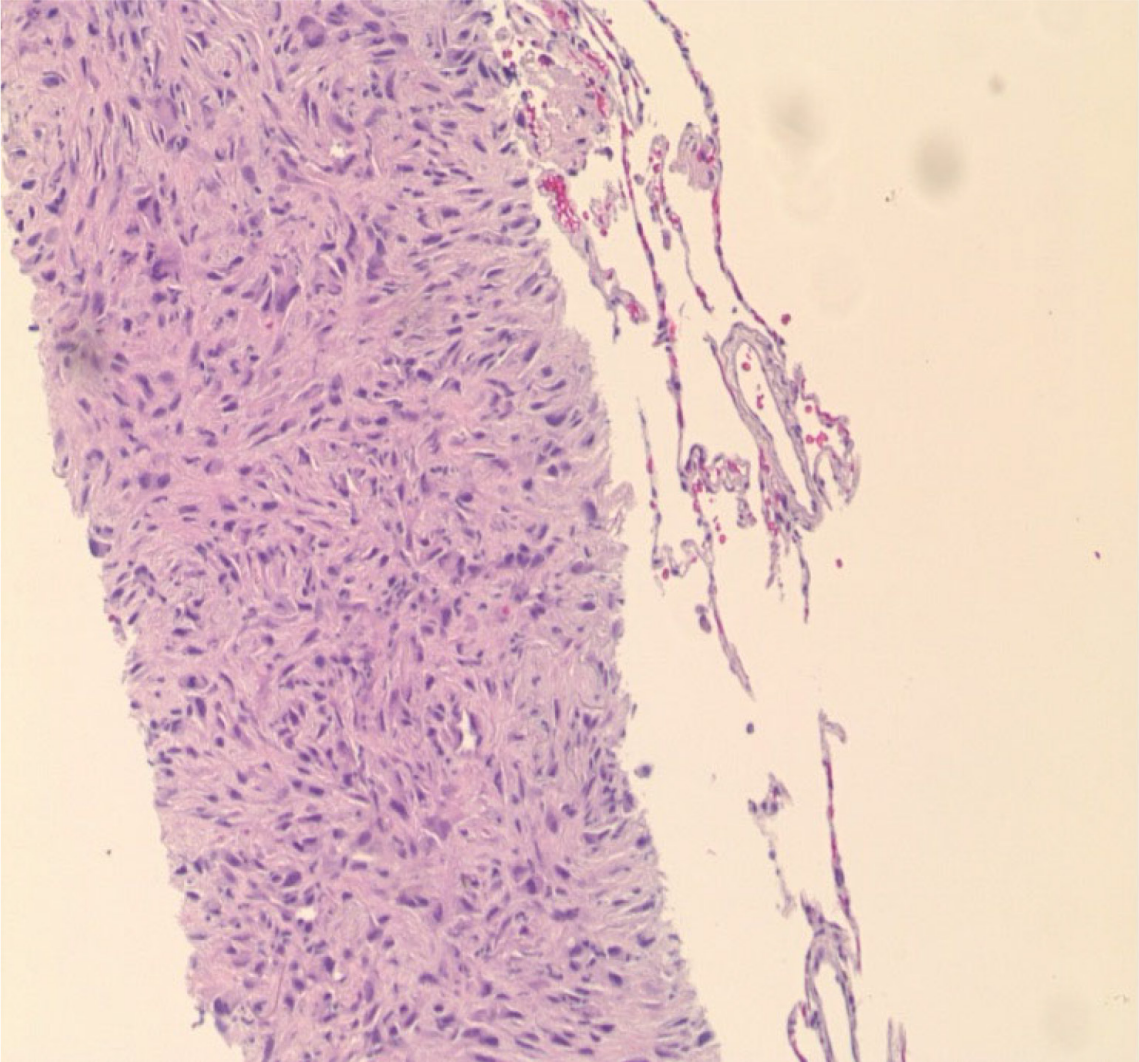
Biopsy of a lung lesion demonstrating spindle cells with ample eosinophilic cytoplasm with obvious cross striations and pleomorphic nuclei with readily apparent mitotic figures.

Further investigation with a CT scan of the chest showed numerous bilateral noncalcified pulmonary parenchymal nodules, with the largest measuring up to 2.2 cm in the left upper lobe, as shown in (Figure 1). Further work‐up for pulmonary nodules, including Histoplasma antigen, cyclic citrullinated peptide antibody, rheumatoid factor, anti‐neutrophilic cytoplasmic antibody, and immunoglobulin subclasses, were within normal limits. The positron emission tomography (PET) scan revealed a small focus of uptake involving the base of the tongue just left to the midline and innumerable fluorodeoxyglucose (FDG)‐avid pulmonary nodules, as shown in (Figure 2). The patient subsequently underwent a CT‐guided biopsy of the lung nodules, which showed spindle cells with ample eosinophilic cytoplasm, obvious cross striations, and pleomorphic nuclei with readily apparent mitotic figures, as shown in Figure 3. Immunoperoxidase staining was positive for actin and desmin, consistent with spindle cell sarcoma favoring LMS. The diagnosis of metastatic LMS was made, with the primary lesion likely being at the base of the tongue based on PET scan FDG activity.

With the suspicion of a paraneoplastic syndrome, antibodies were obtained as mentioned in (Table 1). The jerky movements, in the setting of LMS and positive antibodies, led to the diagnosis of PMD. Subsequently, the patient was prescribed Aripiprazole for abnormal body movement, but it did not help with the symptoms. Given the patient's advanced age and overall poor prognosis, both the patient and the family opted for comfort measures, and the patient was discharged home on hospice care.

**TABLE 1:** Paraneoplastic antibody panel.

Labs	Results	Normal range
CASPR2‐IgG CBA, S	Negative	Negative
LGI1‐IgG CBA, S	Positive	Negative
Amphiphysin Ab, S	Negative	<1:240
AGNA‐1, AGNA‐2, AGNA‐3	Negative	<1:240
PCA‐1, PCA‐2, PCA‐Tr	Negative	<1: 240

Abbreviations: AGNA: anti‐glial/neuronal nuclear antibody; CASPR‐IgG CBA S, contactin‐associated protein‐2 antibody cell‐based assay; LGI1‐IgG CBA, S, Leucine‐rich, glioma inactivated 1 protein IgG cell‐based assay; PCA, Purkinje cell antibody.

## 4 DISCUSSION

LMS is a rare malignant mesenchymal tumor that develops from smooth muscle cells or their precursor cells and eventually develops into smooth muscle cells. Frequent sites of occurrence include the uterine myometrium, gastrointestinal system, retroperitoneum, skin, and subcutaneous tissue. It rarely manifests in the head and neck region owing to the paucity of smooth muscle in this region.^5^ Among the sites of presentation in the head and neck, it usually occurs in the buccal mucosa and rarely affects the tongue.^6^ There have only been a handful of cases of LMS originating primarily in the tongue.

The genetic mechanisms of LMS remain complex, and karyotypic defects involving gains and losses have been observed. The most frequently altered genes in LMS include tumor protein 53 (TP53), ataxia‐telangiectasia mutated (ATM), alpha‐thalassemia/mental retardation, X‐linked (ATRX), epidermal growth factor receptor (EGFR), and retinoblastoma 1 (RB1).^7^ The clinical presentation of LMS is nonspecific and typically manifests as compression or displacement of adjacent organs. The diagnosis is made based on the histological criteria. Histological features include intersecting, sharply marginated fascicles of spindle cells with elongated, hyperchromatic nuclei and abundant eosinophilic cytoplasm with varying degrees of pleomorphism.^4^ It is usually diagnosed with light microscopy, though immunohistochemistry is used for confirmation. In our case, the histological findings of the lung lesion were consistent with LMS. Treatment depends on the stage of presentation, and localized tumors are surgically resected. Metastatic LMS is considered incurable, and the goal of treatment is to control symptoms, decrease tumor bulk, and prolong survival.^4^ In our case, LMS was metastatic and the patient opted for comfort care rather than surgery or chemotherapy.

Paraneoplastic movement disorder is an immune‐mediated non‐metastatic manifestation of cancer that presents as various hypokinetic or hyperkinetic movement abnormalities.^8^ PMD typically presents before the diagnosis of an underlying tumor.^9^ Paraneoplastic movements have been reported commonly in patients with lung cancer, breast cancer, thymoma, lymphoma, and renal cell carcinoma.^10^, ^11^, ^12^ On review of the literature, there have not been any cases of tongue cancer causing PMD. However, our patient's initial presentation was with movements resembling chorea and dystonia. The underlying mechanism of PMD appears to be the expression of autoantigens by the associated neoplasm, which trigger an immune response resulting in autoantibodies that bind to neuronal surface antigens.^13^ The symptoms depend on the regions of the brain affected. Different antibodies have been identified in patients with cancer, such as the collapsin response mediator protein‐5 (CRMP5) antibody in small‐cell lung cancer and testicular cancer. Anti‐Hu, Anti‐Ri, and Anti‐Yo are associated with thymoma, breast cancer, lymphoma, and renal cell cancer.^10^, ^11^, ^14^ We obtained the paraneoplastic autoimmune panel, which showed positive LGI1‐IgG which supports neurological autoimmunity.

LGI1 is a part of the voltage‐gated potassium channel complex and LGI1‐IgG is usually positive in limbic encephalitis.^15^ Anti‐LGI1 encephalitis commonly presents with memory loss, confusion, seizures, and personality changes, and less commonly as a movement disorder.^16^, ^17^ Distinctive faciobrachial dystonic seizures, which are brief and can occur up to 100 times per day, are common with anti‐LGI1 encephalitis and usually precede cognitive symptoms with memory deficits.^13^ Mesial temporal lobe hyperintensity is also observed in the brain MRI late in the disease course of Anti‐LGI1 limbic encephalitis.^18^ In our patient, the MRI finding was normal, which could be explained by the possibility that our patient's MRI was obtained during an earlier phase of the disease course.

The first step in the treatment of PMD is oncological (surgical or systemic) management of the underlying malignancy, followed by immune treatment if required. Immune therapy includes steroids, immunoglobulins, and plasma exchange as first‐line treatments. If the first‐line therapy fails, second‐line options include rituximab, cyclophosphamide, mycophenolate mofetil, and azathioprine.^13^ Symptomatic management can also be considered depending upon the symptoms, with options such as antiseizure medication, antipsychotics, or muscle relaxants. In our case, the patient was prescribed aripiprazole, but it did not significantly help in controlling the symptoms. Given the patient's advanced age and poor overall prognosis, he was discharged to hospice care.

## 5 CONCLUSION

PMD are commonly observed in patients with lung cancer, breast cancer, lymphoma, thymoma, and renal cell cancer, each having specific antibodies associated with the respective cancers. Our case underscores the significance of additional research in this area, emphasizing the need to consider other cancers, such as LMS, in the differential diagnosis of PMD.

## AUTHOR CONTRIBUTIONS

**Pradeep Khanal:** Conceptualization; resources. **Pitambar Khanal:** Writing – original draft. **Sandip Paudel:** Writing – review and editing. **Ashbita Pokharel:** Supervision. **Subodh Chapagain:** Writing – review and editing.

## FUNDING INFORMATION

None.

## CONFLICT OF INTEREST STATEMENT

There is no conflict of interest to be declared.

## CONSENT STATEMENT

Written informed consent was obtained from the patient to publish this report in accordance with the journal's patient consent policy.

## Data Availability

All required information is in the manuscript itself.
